# Severe Noncardiogenic Pulmonary Edema Secondary to Massive Verapamil Overdose and Treatment with Venovenous Extracorporeal Membrane Oxygenation

**DOI:** 10.1155/2020/8842303

**Published:** 2020-12-24

**Authors:** An Ho, Abigail Go, Christopher Barrios, Anthony Scalzo

**Affiliations:** ^1^Division of Pulmonary and Critical Care, Saint Louis University Hospital, St. Louis, Missouri, USA; ^2^Department of Internal Medicine, Saint Louis University Hospital, St. Louis Missouri, USA; ^3^Toxicology, Department of Internal Medicine, Saint Louis University Hospital, St. Louis, Missouri, USA

## Abstract

Calcium channel blocker (CCB) poisoning frequently presents with cardiovascular complications such as cardiogenic shock and arrhythmia. We present a case of massive verapamil overdose causing refractory noncardiogenic pulmonary edema successfully treated with extracorporeal membrane oxygenation. To our knowledge, this is the first case with these features reported in literature. A 27-year-old female patient presented with an overdose of 18,000 mg of verapamil. Her clinical condition deteriorated to severe hypoxic respiratory failure despite being treated with calcium, high-dose insulin, and full invasive ventilation support. She eventually required venovenous extracorporeal membrane oxygenation (VV-ECMO) for three days with full recovery. Large ingestion of verapamil could lead to noncardiogenic pulmonary edema. VV-ECMO might play an important role to support the treatment in severe cases with refractory hypoxia.

## 1. Background

Calcium channel blockers (CCB) like amlodipine and verapamil are fairly common drugs prescribed to patients for hypertension and rhythm control. Though these may not be the most common drugs of choice to overdose, providers should be able to recognize the signs and symptoms of an overdose and quickly start appropriate treatment. The following case illustrates a rare manifestation of calcium channel blocker toxicity which is a noncardiogenic pulmonary edema. This case is also the first case to report the role of venovenous extracorporeal membrane oxygenation (VV-ECMO) as a supportive measure in noncardiogenic pulmonary edema from calcium channel blocker poisoning.

## 2. Case Presentation

**Figure 1:**
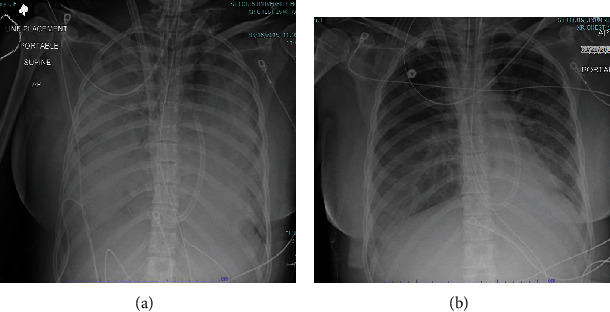
(a) Chest radiograph showed diffuse bilateral alveolar infiltrates on day 3 after the intoxication. (b) Chest radiograph showed significant improvement in bilateral infiltrates on day 5, right before the ECMO decannulation.

A 27-year-old female with a history of depression and asthma presented to the emergency department of an outside hospital one hour after intentionally ingesting 18,000 mg of verapamil. Upon initial presentation, the patient was alert and oriented but hypotensive. Initial treatment included intravenous fluids, activated charcoal, calcium, and glucagon. She had multiple episodes of emesis. The patient remained hypotensive and was started on norepinephrine, dobutamine, and high-dose insulin infusion. She then developed acute respiratory failure with hypoxemia and ultimately required invasive mechanical ventilation. Shortly after intubation, she suffered cardiopulmonary arrest due to pulseless electrical activity (PEA) and received chest compression, atropine, epinephrine, and bicarbonate during resuscitative efforts. Return of spontaneous circulation (ROSC) was achieved after 10 minutes. She was then transferred to our facility for a higher level of care. Upon arrival, she was noted to be hypoxemic with a SpO_2_ of 88% on high PEEP, FiO_2_ 100%, inhaled epoprostenol, and neuromuscular blockade with cisatracurium. Arterial blood gas showed a PaO_2_/FiO_2_ ratio of 80, and chest radiograph revealed diffuse bilateral airspace opacities consistent with severe acute respiratory distress syndrome (ARDS). The trial of high-dose diuretics failed to improve oxygenation, and transthoracic echocardiogram revealed adequate right and left ventricular function and normal estimated end-diastolic filling pressure with an *e*/*e*′ ratio of 8. Hence, the patient was placed on VV-ECMO. Vasopressor and neuromuscular blocker were quickly weaned off after 24 hours. The patient had initially required a sweep gas of 5 L but was weaned down to 1 L with the initiation of the ECMO blender. After three days on VV-EMCO, the patient was decannulated off ECMO.  Figure 1 demonstrated the improvement in chest radiograph of the patient on day 3 and day 5. She was extubated on day 6. The patient was eventually discharged from the intensive care unit without further event. The duration of the patient's stay was 7 days in the intensive care unit and 3 more days in the general medical ward.

## 3. Outcome and Follow-Up

This young, female patient was successfully treated for acute CCB overdose in the medical ICU. She was weaned completely off oxygen and did not have any other respiratory symptoms at discharge from the general medical floor. She required an extended stay of three more days at a psychiatric ward for her suicide attempt and depression.

## 4. Discussion

An overdose of verapamil, a nondihydropyridine CCB, could lead to life-threatening cardiovascular collapse and arrhythmias. In most cases of toxicity, the primary objective of treatment is establishing hemodynamic stability. Proposed treatments include gastrointestinal decontamination, intravenous fluid, vasopressors, calcium, high-dose insulin therapy, and lipid emulsion therapy [1]. Calcium infusions are thought to increase the extracellular concentration and then cause calcium influx through the L-type calcium channels that remain unblocked [2]. Animal studies have shown improvement in mortality and hemodynamics, but a human case series show inconsistent benefits [3]. High-dose insulin (HDI) with glucose has also emerged as a cornerstone of treatment [4]. Cardiac myocytes preferentially use free fatty acids for their energy substrate. CCB toxicity causes a shift from this to using carbohydrates as the main substrate. Though the mechanism is not fully understood, HDI therapy may overcome this metabolic deficit [2, 4]. HDI also has a positive ionotropic effect that is not catecholamine mediated [1].

Our case represents a severe acute respiratory distress syndrome which was nonresponsive to the first-line management. Noncardiogenic pulmonary edema occurs when there is radiographic evidence of alveolar fluid collection in the setting of a pulmonary artery wedge pressure ≤ 18 mmHg. Acute respiratory distress syndrome is the most recognized form of noncardiogenic pulmonary edema. Other etiologies include high-altitude pulmonary edema, neurogenic pulmonary edema, opioid and salicylate overdose, and transfusion-related acute lung injury, among other causes [5]. Noncardiogenic pulmonary edema due to CCB is rare and in a few published case reports worldwide, and most of the patients survived with first-line therapies [6–8]. As lipophilic, nondihydropyridine CCBs, verapamil and diltiazem are known to be more cardiotoxic than dihydropyridine CCBs like amlodipine and nifedipine. As such, the pulmonary edema was previously thought to be due to the negative inotropy from CCB [8]. However, the more recent theory postulates that the mechanism involves precapillary vasodilation which results in excessive pulmonary capillary transudation [9].

Our case represents severe refractory ARDS due to verapamil which was nonresponsive to the first-line management and mechanical ventilation. ECMO as a supportive treatment for drug poisoning was rarely reported to the American College of Medical Toxicology investigator consortium [10]. Only 0.0004% of all drug poisoning cases from 2010 to 2013 reported using ECMO [10] with a favorable survival rate from 76% to 80% [10, 11]. Duration of ECMO support was 4 hours to 4 days [10]. The role of venoarterial extracorporeal membrane oxygenation (VA-ECMO) support has been suggested for cardiogenic shock due to CCB toxicity with less than 10 cases reported worldwide [12, 13]. The outcome was good with recovery of all cases in a case series using VA-ECMO by Vignesh et al. [13]. To our knowledge, our patient is the first case of noncardiogenic pulmonary edema that was successfully treated with VV-ECMO assistance.

## 5. Learning Points/Take Home Messages

- Providers should be aware of signs and symptoms of CCB toxicity as well as proposed treatments as these medications are commonly prescribed and are therefore accessible to a large segment of the population
- CCB toxicity is typically treated with hemodynamic support, high-dose insulin and glucose (HDI), and lipid emulsion therapy though efficacy has been inconsistent
- Noncardiogenic pulmonary edema is a rare but possible effect of CCB toxicity. When therapies including first-line treatment for calcium channel blocker toxicity and mechanical ventilation fail, advanced life support measures like ECMO can be utilized to further support patients
